# AHA1 upregulates IDH1 and metabolic activity to promote growth and metastasis and predicts prognosis in osteosarcoma

**DOI:** 10.1038/s41392-020-00387-1

**Published:** 2021-01-20

**Authors:** Diwei Zheng, Weihai Liu, Wenlin Xie, Guanyu Huang, Qiwei Jiang, Yang Yang, Jiarong Huang, Zihao Xing, Mengling Yuan, Mengning Wei, Yao Li, Junqiang Yin, Jingnan Shen, Zhi Shi

**Affiliations:** 1 Department of Cell Biology & Institute of Biomedicine, National Engineering Research Center of Genetic Medicine, MOE Key Laboratory of Tumor Molecular Biology, Guangdong Provincial Key Laboratory of Bioengineering Medicine, College of Life Science and Technology, Jinan University, Guangzhou, Guangdong 510632 China; 2 Department of Musculoskeletal Oncology, the First Affiliated Hospital of Sun Yat-sen University, Guangzhou, Guangdong 510080 China; 3 Guangdong Provincial Key Laboratory of Orthopedics and Traumatology, Guangzhou, Guangdong 510080 China; 4 Department of Pathology, the Seventh Affiliated Hospital of Sun Yat-sen University, Shenzhen, Guangdong 518017 China

**Keywords:** Bone cancer, Oncogenes

## Abstract

Osteosarcoma (OS) is the most common primary malignant bone tumor in children and adolescents. Although activator of HSP90 ATPase activity 1 (AHA1) is reported to be a potential oncogene, its role in osteosarcoma progression remains largely unclear. Since metabolism reprogramming is involved in tumorigenesis and cancer metastasis, the relationship between AHA1 and cancer metabolism is unknown. In this study, we found that AHA1 is significantly overexpressed in osteosarcoma and related to the prognosis of osteosarcoma patients. AHA1 promotes the growth and metastasis of osteosarcoma both in vitro and in vivo. Mechanistically, AHA1 upregulates the metabolic activity to meet cellular bioenergetic needs in osteosarcoma. Notably, we identified that isocitrate dehydrogenase 1 (IDH1) is a novel client protein of Hsp90-AHA1. Furthermore, the IDH1 protein level was positively correlated with AHA1 in osteosarcoma. And IDH1 overexpression could partially reverse the effect of AHA1 knockdown on cell growth and migration of osteosarcoma. Moreover, high IDH1 level was also associated with poor prognosis of osteosarcoma patients. This study demonstrates that AHA1 positively regulates IDH1 and metabolic activity to promote osteosarcoma growth and metastasis, which provides novel prognostic biomarkers and promising therapeutic targets for osteosarcoma patients.

## Introduction

Osteosarcoma is a deadly primary bone malignancy that affects children and adolescents.^1–3^ In the last few years, neoadjuvant chemotherapy and surgery have shown tremendous progress in reducing the tumor burden, but the 5-year survival of osteosarcoma patients with metastasis and recurrence after conventional therapy remains low, <20%.^4,5^ Moreover, administration of cisplatin as a single-drug chemotherapy for osteosarcoma patients might lead to ototoxicity.^6^ Thus, it is indispensable to develop molecular targeted agents with high tumor specificity to address these problems.

Heat-shock protein 90 (Hsp90), a molecular chaperone, is essential for eukaryotic cell survival and has been highly conserved during evolution.^7^ Hsp90 consists of three domains, namely the N, middle, and C domain,^8^ and regulates the balance of stabilization, activation, and degradation of client proteins, including numerous transcription factors and kinases, such as C-RAF, CDC37, CDK4, and ERBB2, which are involved in signaling pathways crucial for the development and maintenance of a malignant phenotype.^9^

AHA1, as a co-chaperone of Hsp90, generally enhances the function of Hsp90 through the hydrolyzation of adenosine triphosphate (ATP) to stimulate the ATPase activity of Hsp90.^10–12^ AHA1 promotes effective folding of Hsp90-dependent client proteins, such as steroid receptors and many kinases involved in cellular signaling, including FKBP52 and p23/Sba1.^10,13^ In addition, AHA1 regulates the activity of Hsp90 and its client proteins to affect tumor progression through post-translational modifications.^14^

Cancer cells always have a huge demand for energy due to the fast proliferation and highly invasive behaviors. Therefore, cancer cells tend to maintain cellular energy homeostasis through metabolism rewiring. To achieve this goal, cancer cells overexpress some oncogenes to upregulate metabolic activity, such as MYC^15^ and KRAS.^16^ Cancer cells also exhibit enhanced glucose consumption and lactate production even in the presence of abundant oxygen, which is known as the Warburg effect and related to proliferation, metastasis, and drug resistance in cancers. As a co-chaperone of Hsp90, AHA1 may also regulate some client proteins that are involved in cellular metabolism. However, the relationship between Hsp90-AHA1 and metabolism reprogramming in osteosarcoma remains unknown.

In this study, we found that the expression level of AHA1 was significantly higher in osteosarcoma and high AHA1 expression was associated with the poor prognosis of osteosarcoma patients. Further studies suggested that AHA1 promoted the growth and metastasis of osteosarcoma both in vitro and in vivo by upregulating IDH1 and metabolic activity. Moreover, we identified that Hsp90-AHA1 directly interacts with IDH1 and positively regulates its level. Notably, there is a positive co-expression relationship between AHA1 and IDH1 in osteosarcoma tissues. In conclusion, this study demonstrates the oncogenic role of AHA1 in osteosarcoma and provides potential therapeutic targets and prognostic factors for osteosarcoma patients.

## Results

### Upregulation of AHA1 in osteosarcoma is correlated with lung metastasis and poor prognosis

**Fig. 1:**
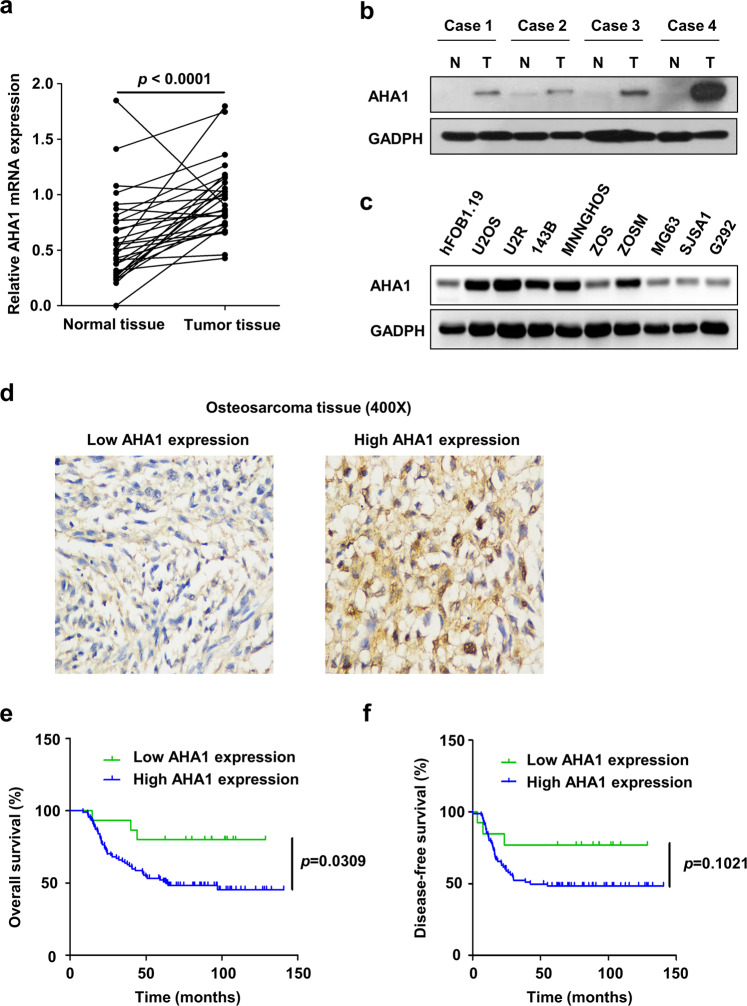
Upregulation of AHA1 in osteosarcoma is correlated with poor prognosis. **a** RT-qPCR analysis of relative AHA1 expression in osteosarcoma tissues and adjacent normal tissues. The data were statistically analyzed using paired *t*-test. AHA1 protein expression in tissues (osteosarcoma and adjacent normal tissues) (**b**) and cells (osteoblast and osteosarcoma cells) (**c**) was detected by WB. **d** The representative images of low AHA1 expression or high AHA1 expression in osteosarcoma tissues were shown. **e**, **f** Kaplan–Meier analysis of overall and disease-free survival for osteosarcoma patients with high or low AHA1 expression

To explore the expression level of AHA1 in osteosarcoma, AHA1 expression was detected in osteosarcoma and paired normal tissues through RT-qPCR. Compared with adjacent normal tissues, the mRNA level of AHA1 was significantly upregulated in osteosarcoma tissues (*p* < 0.0001, Fig. 1a). And the protein level of AHA1 was also higher in osteosarcoma tissues when compared with adjacent normal tissues (Fig. 1b). As shown in Fig. 1c, AHA1 was more highly expressed in some human osteosarcoma cell lines (U2OS, U2R, 143B, MNNGHOS, ZOSM) compared with normal bone cell lines (hFOB1.19). Moreover, we found that the expression of AHA1 was associated with lung metastasis and death but not with age, gender or primary tumor location (Supplementary Fig. S1 and Supplementary Table S1). A Kaplan–Meier analysis revealed that high AHA1 expression was correlated with poor overall survival (*p* = 0.0309) and disease-free survival (*p* = 0.1021) (109 patients, Fig. 1d–f). Briefly, these findings indicate that the upregulation of AHA1 in osteosarcoma tissues is correlated with lung metastasis and poor prognosis.

### AHA1 is important for the growth, migration, and drug resistance of osteosarcoma cells in vitro

**Fig. 2:**
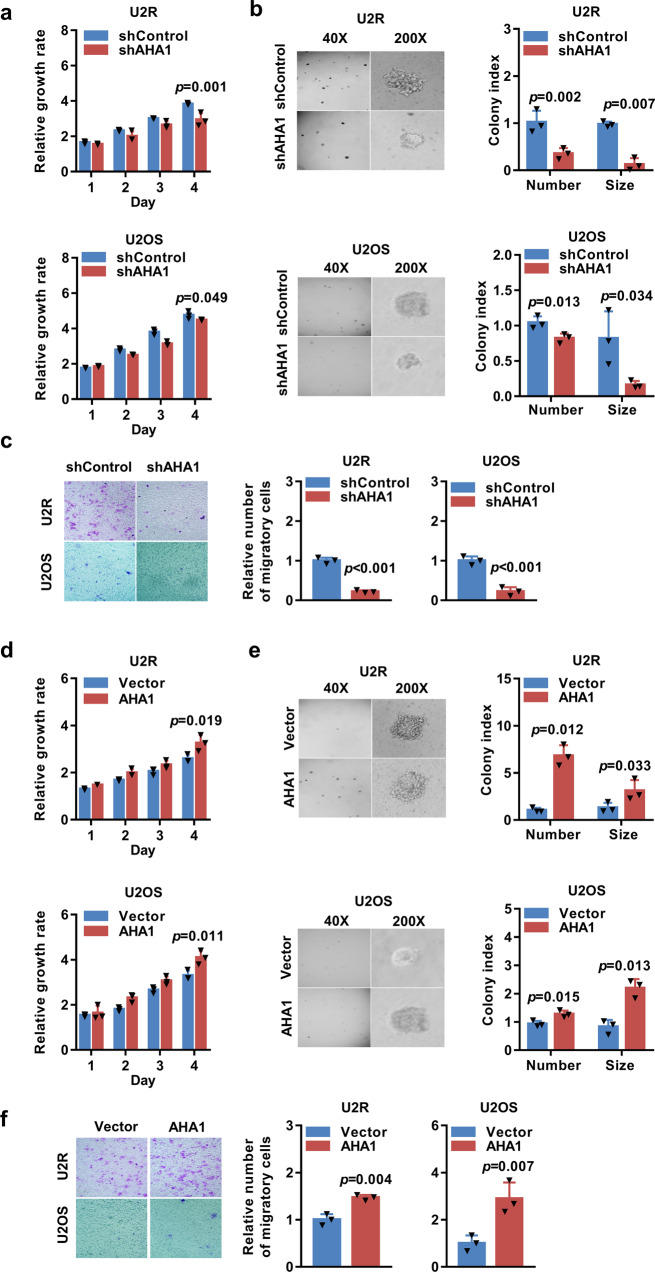
AHA1 is important for the growth and migration of osteosarcoma cells in vitro. **a**, **d** Proliferation was examined by MTT assay. **b**, **e** Time course of anchorage-independent colony formation of U2R and U2OS cells, and representative images of colony growth after 2 weeks are shown. **c**, **f** Cells were cultured in the Transwell® cell culture insert. The number of cells that traversed the filters was counted, and triplicate samples were analyzed. **a**–**f** Data are shown as mean ± SD of three independent cultures

To investigate the function of AHA1 in osteosarcoma, human osteosarcoma cell line U2OS and the related methotrexate-resistant cell line U2R were used for AHA1 knockdown or overexpression. We then verified that the expression of AHA1 was successfully downregulated or upregulated in the cells (Supplementary Fig. S2a, b). The downregulation of AHA1 significantly suppressed cell proliferation in the U2R and U2OS cells (Fig. 2a). Moreover, cells stably expressing shAHA1 formed smaller and fewer colonies than the shRNA control cells (Fig. 2b). The wound healing and transwell assays indicated that the migration ability of the cells stably expressing shAHA1 significantly decreased when compared with the control cells (Fig. 2c and Supplementary Fig. S2c). Phalloidin staining of F-actin showed that AHA1 knockdown markedly suppressed the formation of thick fibers and focal adhesion points in U2R and U2OS cells, confirming that AHA1 exerts a promoting effect in cell migration (Supplementary Fig. S2e). On the contrary, we observed that the AHA1-overexpressing cells had a significantly faster growth rate and an enhanced ability to grow in soft agar when compared with the control vector-transfected cells (Fig. 2d, e). Moreover, we found that the migration ability of AHA1-overexpressing cells dramatically increased, as demonstrated through wound healing and transwell assays (Fig. 2f and Supplementary Fig. S2d). In addition, phalloidin staining of F-actin showed that the ectopic expression of AHA1 significantly promoted the formation of thick fibers and focal adhesion points in U2R and U2OS cells (Supplementary Fig. S2f). Therefore, AHA1 overexpression promotes the migration of U2R and U2OS cells.

Additionally, the sensitivity of U2R and U2OS cells to Hsp90 inhibitor 17AAG or ganetespib at certain concentrations was enhanced by the downregulation of AHA1 (Supplementary Fig. S3a). However, AHA1 overexpression did not affect the sensitivity of the U2OS or U2R cells to 17AAG or ganetespib (Supplementary Fig. S3b). Similar published data demonstrated that the overexpression of AHA1 did not influence the sensitivity to 17AAG, but excessive AHA1 could stimulate increases in the C-RAF, *p*-MEK1/2, and *p*-ERK1/2 levels without changing the total levels of these proteins.^9^

Furthermore, we found reintroduction of AHA1 could rescue the effect of AHA1 knockdown on the growth and migration of osteosarcoma cells (Supplementary Fig. S4a-d), which further confirms the knockdown specificity and the oncogenic role of AHA1. Collectively, these data suggest that AHA1 positively affects the growth, migration and drug resistance of osteosarcoma cells in vitro.

### AHA1 knockdown significantly suppresses the growth and metastasis of osteosarcoma cells in vivo

**Fig. 3:**
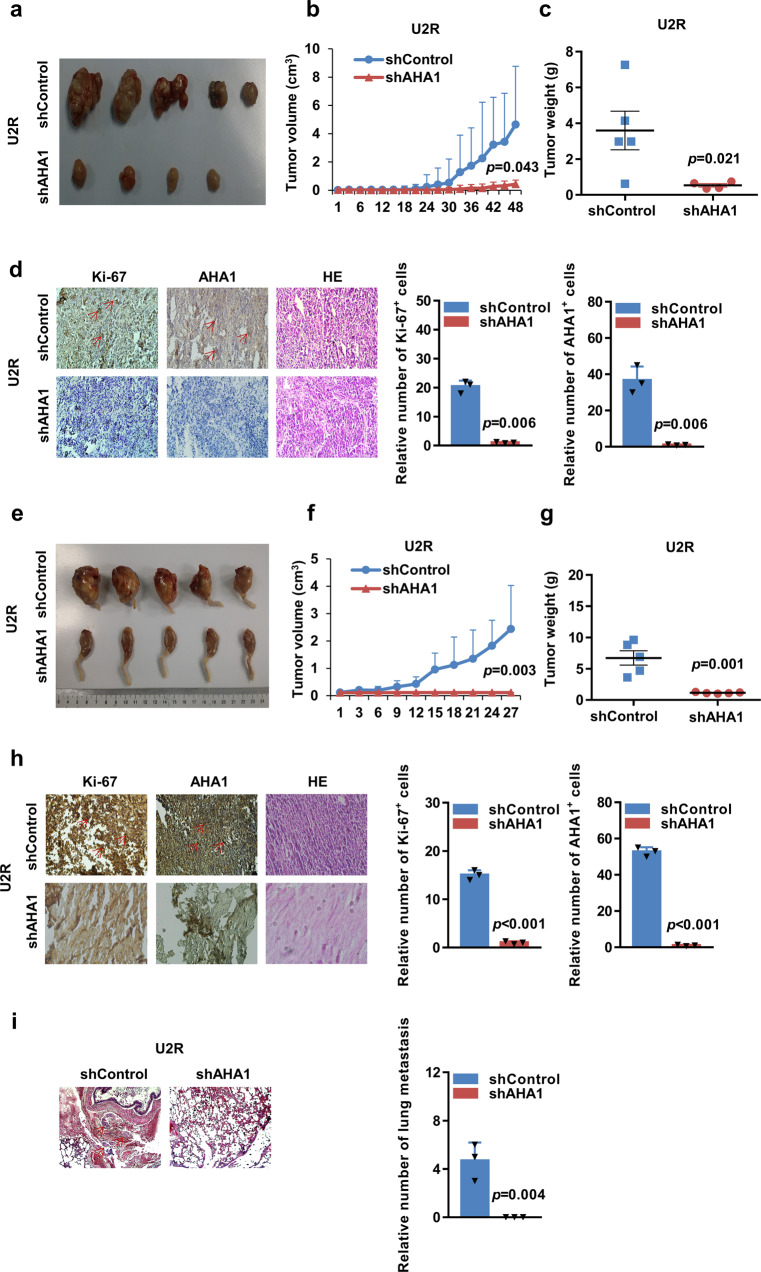
AHA1 knockdown significantly suppresses the growth and metastasis of osteosarcoma cells in vivo. Each nude mouse was inoculated subcutaneously (2 × 10^6^ in 100 μl of medium) or injected into the tibial medullary cavity (2 × 10^6^ in 25 μl of medium) with U2R-shControl and U2R-shAHA1 cells. The original tumors (**a**, **e**), tumor volume (**b**, **f**), and tumor weight (**c**, **g**) are shown. Representative images of H&E, Ki-67, and AHA1 staining of U2R-shControl and U2R-shAHA1 tumors (**d**, **h**) are also presented. **i** The nude mice were injected with 2 × 10^6^ U2R-shControl or U2R-shAHA1 cells in 200 μl of medium into their tail veins. Representative images of H&E staining of the lungs and the analysis of the number of lung metastasis are shown. The values presented are the mean ± SD for each group

To prove that AHA1 regulates the growth of osteosarcoma cells in vivo, we subcutaneously injected U2R cells stably expressing shAHA1 or shControl into BALB/c nude mice to assess their tumorigenicity in vivo. As shown in Fig. 3a–c, knockdown of AHA1 significantly inhibited the growth of U2R tumors, as demonstrated by reductions in tumor volume and tumor weight. And IHC staining showed that AHA1 knockdown significantly decreased the number of Ki-67^+^ proliferating cells in U2R tumors (Fig. 3d). To further investigate the effect of AHA1 on tumorigenesis of osteosarcoma cells in vivo, we established an orthotopic model by injecting U2R cells stably expressing shAHA1 or shControl into tibial medullary cavity of the right leg of BALB/c nude mice. Remarkably, the U2R-shControl cells developed much larger and heavier tumors than the U2R-shAHA1 cells (Fig. 3e–g). Additionally, representative IHC staining images showed that the numbers of Ki-67^+^ and AHA1^+^ cells obviously decreased in U2R-shAHA1 tumors when compared with U2R-shControl tumors (Fig. 3h). To study the role of AHA1 in lung metastasis of osteosarcoma, we subsequently built a model of lung metastasis through tail vein injection. H&E staining revealed no metastasis in the U2R-shAHA1 group, whereas metastasis was observed in U2R-shControl group (Fig. 3i). Thus, AHA1 knockdown significantly suppresses the growth and metastasis of osteosarcoma in vivo.

### AHA1 is critical for metabolic activity and regulates IDH1 level through interaction

**Fig. 4:**
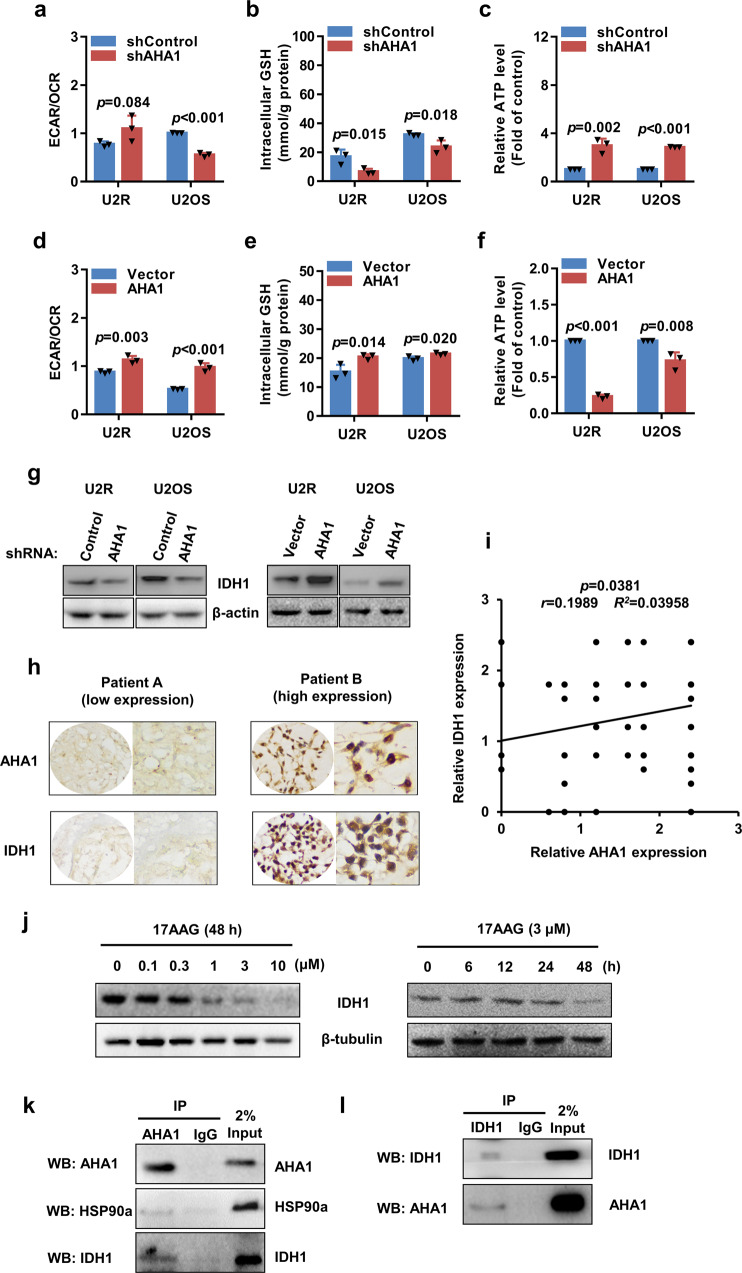
AHA1 is critical for metabolic activity and regulates IDH1 level through interaction. **a**, **d** ECAR/OCR levels of U2R and U2OS with AHA1 knockdown or overexpression were analyzed with an XFe96 Analyzer (Seahorse Bioscience). **b**, **c**, **e**, **f** Intracellular glutathione and ATP levels of U2R and U2OS with AHA1 knockdown or overexpression are shown. **g** IDH1 protein level in U2R and U2OS cells with AHA1 knockdown or overexpression was detected. **h** Representative IHC images of AHA1 and IDH1 expression in the same osteosarcoma patients. **i** Pearson’s correlation scatter plot of the expression levels of AHA1 and IDH1 in human osteosarcoma tissues. **j** IDH1 protein level after incubation with different concentrations of 17AAG (0, 0.1, 0.3, 1, 3, 10 μM) or incubation for various durations (0, 6, 12, 24, 48 h) was detected. **k**, **l** Interaction between endogenous AHA1 and IDH1 was detected by co-immunoprecipitated (co-IP). **a**–**f** Data are shown as mean ± SD of three independent cultures

Metabolism reprogramming is playing an important role in tumorigenesis and cancer metastasis.^17^ To understand the mechanisms underlying the tumorigenic function of AHA1 in osteosarcoma, we examined whether AHA1 affects the metabolic activity of osteosarcoma cells. When compared with control cells, knockdown of AHA1 resulted in significant reductions in extracellular acidification rate/oxygen consumption rate (ECAR/OCR). In contrast, overexpression of AHA1 significantly increased ECAR/OCR (Fig. 4a, d). This suggests AHA1 level is critical for glycolysis. Moreover, glucose consumption and lactate production were lower in AHA-knockdown osteosarcoma cells and higher in AHA1-overexpressing osteosarcoma cells when compared with controls, which further confirms the important role of AHA1 in glycolysis (Supplementary Fig. S5a-d). Furthermore, AHA1 knockdown significantly inhibited both intracellular glutathione (GSH) biosynthesis and ATP consumption, while AHA1-overexpressed cells showed increased intracellular glutathione biosynthesis and ATP consumption (Fig. 4b, c, e, f), indicating AHA1 positively regulates biomass biosynthesis and energy consumption in osteosarcoma cells.

Tumor cells always upregulate the critical enzymes to fuel the energetic and biomass demand for cell growth. Our findings suggest that AHA1 is critical for the metabolic activity of osteosarcoma cells. However, whether AHA1 can regulate the enzymes for cellular metabolism has never been reported. Therefore, we aimed to investigate the relationship between AHA1 and the critical enzymes that are involved in glycolysis and tricarboxylic acid cycle (TCA cycle). Interestingly, we found AHA1 knockdown reduced the level of some metabolic enzymes, among which IDH1 decreased most significantly. To further confirm the role of AHA1 in the regulation of IDH1, protein level of IDH1 in U2R and U2OS cells with AHA1 overexpression was detected. Consistently, AHA1 overexpression dramatically increased IDH1 in osteosarcoma cells (Fig. 4g). To further confirm the relationship between AHA1 and IDH1, the level of AHA1 and IDH1 were detected by IHC in 109 cases of osteosarcoma tissues. And the result indicated that IDH1 protein expression was positively correlated with the AHA1 protein expression (Fig. 4h, i and Supplementary Table S2). Moreover, we found that 17AAG could downregulate IDH1 protein level in both dose- and time-dependent manners (Fig. 4j). The two domains of AHA1 could bind to the middle and N-terminal domains of Hsp90 to activate the ATPase function of Hsp90.^18^ And Hsp90-AHA1 complex could directly interact with their client proteins and further regulate these proteins.^14^ Therefore, we hypothesized that IDH1 may be a novel client protein of Hsp90-AHA1 complex. To validate this hypothesis, we detected the interaction between endogenous AHA1 and IDH1 through Co-IP. As shown in Fig. 4k, AHA1 directly interacted with Hsp90α and IDH1 but not with control immunoglobulin G (IgG). Furthermore, we found that IDH1 could directly interact with AHA1 (Fig. 4l). Taken together, these data indicate that AHA1 significantly increases the metabolic activity of osteosarcoma cells. Moreover, Hsp90-AHA1 complex interacts with IDH1 and regulates IDH1 level to affect the metabolic activity of osteosarcoma cells.

### IDH1 overexpression partially reverses the suppressive effects of AHA1 knockdown on the growth and migration of osteosarcoma cells

**Fig. 5:**
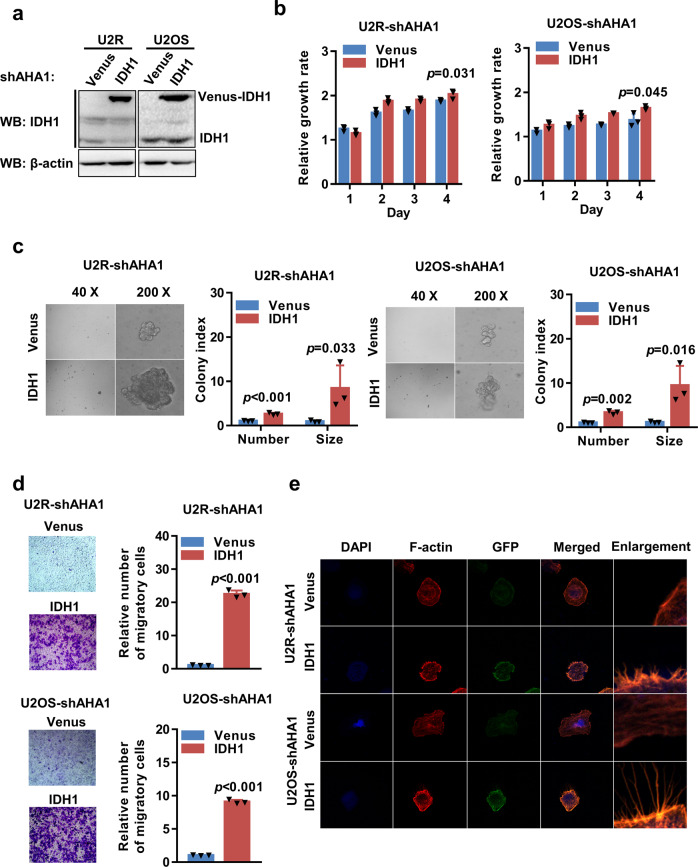
IDH1 overexpression partially reverses the suppressive effects of AHA1 knockdown on the growth and migration of osteosarcoma cells. **a** WB analysis of IDH1 protein expression in the indicated cells. β-actin was used as the loading control. **b** The proliferation of the indicated cells was determined by MTT assay. **c** Time course of anchorage-independent colony formation of U2R and U2OS cells, and representative images of colony growth after 2 weeks are shown. **d** Representative images of cell migration obtained with the Transwell assay are shown, and the levels of cell migration were quantified. **e** Cells were subjected to F-actin (red) and DAPI (blue) staining and analyzed under an immunofluorescence microscope. Data are presented as mean ± SD of three independent experiments. Student’s *t*-test was used for the statistical analysis

To test the role of IDH1 in the oncogenic function of AHA1, AHA1-knockdown cells were transfected with lentivirus expressing IDH1, which successfully upregulated the expression of IDH1 protein in the cells (Fig. 5a). The proliferation of cells with IDH1 overexpression significantly increased when compared with the control vector-transfected cells (Fig. 5b). Moreover, overexpression of IDH1 increased the size and number of colony formation in the U2R- and U2OS-shAHA1 cells (Fig. 5c). The migration ability of the shAHA1-transfected cells overexpressing IDH1 was enhanced when compared with the control vector-transfected cells (Fig. 5d). Similarly, compared with the control vector-transfected cells, overexpression of IDH1 promoted the growth of microfilaments, as demonstrated by F-actin staining (Fig. 5e). Furthermore, we found IDH1 knockdown enhanced the effect of AHA1 knockdown on the growth and migration of osteosarcoma cells (Supplementary Fig. S6a-d). And IDH1 knockdown also increased the sensitivity to both 17AAG and Ganetespib in osteosarcoma cells with AHA1 knockdown (Supplementary Fig. S6e). Collectively, these data indicate that restoration of IDH1 expression partially reverses the suppressive effects of AHA1 knockdown on the growth and migration of osteosarcoma cells.

### Upregulation of IDH1 in osteosarcoma is associated with poor prognosis of osteosarcoma patients

**Fig. 6:**
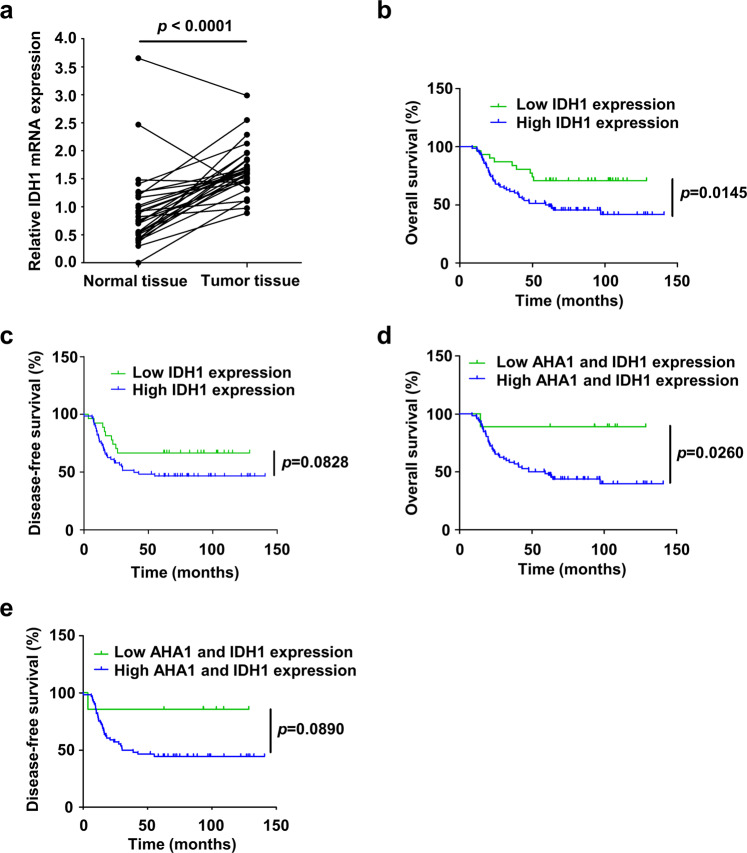
Upregulation of IDH1 in osteosarcoma is associated with poor prognosis of osteosarcoma patients. **a** RT-qPCR analysis of relative IDH1 expression in osteosarcoma tissues and adjacent normal tissues. The data were statistically analyzed using paired *t*-test. **b**–**e** Kaplan–Meier analysis of overall and disease-free survival for osteosarcoma patients with high or low expression of AHA1 and IDH1

To further determine the clinical association of IDH1, the IDH1 expression was detected in osteosarcoma and paired normal tissues through RT-qPCR. Compared with adjacent normal tissues, the mRNA level of IDH1 was significantly upregulated in osteosarcoma tissues (Fig. 6a). Moreover, we found that IDH1 level was associated with death but not age, gender or primary tumor location (Supplementary Fig. S7 and Supplementary Table S1). Notably, Kaplan–Meier analysis revealed that high IDH1 protein level was associated with poor overall survival (*p* = 0.0145) and disease-free survival (*p* = 0.0828) (109 patients, Fig. 6b, c), which suggests osteosarcoma patients with lower IDH1 protein expression exhibited better prognosis. Furthermore, osteosarcoma patients with lower protein expression of both AHA1 and IDH1 exhibited better prognosis (Fig. 6d, e), even though the difference of disease-free survival were not statistically significant due to the inadequate number of cases. More importantly, multivariate analyses revealed that high AHA1 and IDH1 expression could be independent predictors of poor prognosis (Supplementary Table S3). In conclusion, these results indicate that AHA1 and IDH1 might have oncogenic functions and potential values in osteosarcoma prognosis prediction.

## Discussion

Accumulating evidence has shown that Hsp90 chaperone machinery is a key regulator of cellular process by interacting with the client proteins, assisting their correct folding, and increasing their stability as well as their activity.^19^ Among the client proteins of Hsp90, there are a lot of proteins that are involved in cancer progression, which makes Hsp90 a promising target for anti-cancer therapy. The novel specific targeting therapies are required for osteosarcoma therapies, particularly in metastatic or relapsed osteosarcoma patients.^5^ Studies in recent years suggest targeting Hsp90 may be a promising strategy for the treatment of osteosarcoma. The Hsp90 inhibitor geldanamycin (GA) can suppress proliferation and induce apoptosis of KTHOS cells,^20^ and the Hsp90 inhibitor STA-1474 can trigger caspase-3 and restrain *p*-Met/Met and *p*-Akt/Akt in osteosarcoma xenografts.^21^ And the study of Benjamin Ory et al. also shows that Hsp90 is an effective drug target for osteosarcoma by applying a specific Hsp90 inhibitor PF4942847 to treat osteosarcoma in preclinical models.^22^ However, inhibition of Hsp90 may cause some toxic side-effects based on some results in both preclinical and clinical studies.^23,24^ Therefore, we aim to seek some alternative targets on the base of Hsp90 chaperone machinery.

AHA1, as a co-chaperone of Hsp90, stimulates Hsp90 ATPase activities to stabilize client proteins and enhance their functions.^13,25^ However, the role of AHA1 in osteosarcoma and the mechanism remain largely unknown. In this study, we observed the overexpression of AHA1 in osteosarcoma, which is associated with the poor prognosis of osteosarcoma patients. Further study demonstrated that AHA1 could positively affect the growth and metastasis of osteosarcoma cells both in vivo and in vitro, indicating that AHA1 might be a novel oncogene in osteosarcoma, which is consistent with the previous studies of Hsp90 inhibitors in osteosarcoma. Targeting AHA1 may block the effect of Hsp90 on the client proteins and subsequently inhibit the ability of proliferation, migration and invasion in osteosarcoma cells. And the client proteins that are affected by targeting AHA1 may play an important role in osteosarcoma progression.

Compared to normal cells, most tumor cells are always facing a more stressed microenvironment due to the fast growth, quick energy consumption, and insufficient nutrition supply. To meet the huge demand for energy and biomass generation, tumor cells tend to enhance their metabolic activity through upregulating the level and activity of enzymes that are critical for cellular metabolism.^26,27^ In this study, we demonstrated that AHA1 facilitates the metabolic activity of osteosarcoma cells, including the consumption of glucose and ATP, production of lactate and glutathione, which is associated with glycolysis and TCA cycle. Surprisingly, we found AHA1 positively regulates ATP consumption, which suggests AHA1 may favor biomass production over ATP generation. As osteosarcoma is highly malignant and aggressive, the newly discovered role of AHA1 in cellular metabolism regulation may partly explain why AHA1 could promote the growth and metastasis of osteosarcoma. More importantly, we identified for the first time that IDH1 is a novel client protein of Hsp90-AHA1 complex, which supports the pleiotropic functions of Hsp90. We found that Hsp90-AHA1 directly interacts with IDH1 and regulates the level of IDH1. Consistently, the Hsp90 inhibitor 17AAG significantly reduced IDH1 at the protein level. Notably, the positive co-expression relationship between AHA1 and IDH1 was further confirmed in osteosarcoma tissues. Overexpression of IDH1 partially reversed the suppressive effects of AHA1 knockdown on osteosarcoma cells. IDH1 is a critical enzyme of TCA cycle that catalyzes the conversion of isocitrate to alpha-ketoglutarate.^28^ And IDH1/2 genes are mutated in some cancers, including gliomas, osteosarcoma, and AML.^29^ The present study revealed the overexpression of IDH1 in osteosarcoma and high IDH1 protein level was correlated with poor prognosis of osteosarcoma patients. Similarly, high IDH1 expression has been associated with a poor prognosis of CN-AML.^30^ Consequently, targeting IDH1 might become a practical strategy in anti-cancer therapy. In fact, the IDH1 inhibitor AG-120 has been investigated in clinical studies with promising results.^31^ In addition, we found that a combination of AHA1 and IDH1 expression in osteosarcoma patients can also predict the prognosis. In the future, more osteosarcoma tissue samples should be studied to confirm the potential value of AHA1, IDH1, and the combination of them in the diagnosis and prognosis prediction of osteosarcoma patients.

In conclusion, this study shows AHA1 promotes the growth and metastasis of osteosarcoma by upregulating IDH1 and metabolic activity. Moreover, AHA1 and IDH1 could serve as effective diagnostic and prognostic biomarkers for osteosarcoma patients. Therefore, our findings provide novel insights into the oncogenic role of AHA1 in osteosarcoma and promising therapeutic targets for osteosarcoma patients.

## Materials and methods

### Patients and specimens

A total of 109 cases of osteosarcoma tissues were obtained from osteosarcoma patients who underwent surgical excision at the First Affiliated Hospital of Sun Yat-sen University, Guangzhou, China. The study was approved by the ethics committee of the First Affiliated Hospital of Sun Yat-sen University.

### Cell culture and reagents

The human osteosarcoma cell lines U2OS, HOS, 143B, MNNGHOS, ZOS, ZOSM, MG63, SJSA1, G292, the normal osteoblast cell line hFOB1.19, and embryonic kidney cell line HEK293T were cultured in Dulbecco’s modified Eagle’s medium (DMEM) with 10% fetal bovine serum (FBS), penicillin (100 U/ml) and streptomycin (100 ng/ml) at 37 °C with 5% CO_2_ in a humidified incubator. U2OS/MTX300 cells (U2R), a methotrexate-resistant derivative of the U2OS human osteosarcoma cell line, were provided by Dr. M. Serra (Istituti Ortopedici Rizzoli, Bologna, Italy) and were continuously cultured in the presence of 300 μg/L MTX.^32^ Methylthiazolyldiphenyl-tetrazolium bromide (MTT), Agarose M, hematoxylin and other chemicals were purchased from Sangon Biotech (Shanghai). 17AAG and ganetespib were purchased from ApexBio. Anti-AHA1 (A2617) was purchased from Abclonal. Anti-IDH1 (D121821) and anti-β-actin (D110007) antibodies were procured from Sangon. Anti-Ki-67 (RLT2467) antibody and anti-Hsp90α (RLT2257) antibody were purchased from Ruiying Biological. Anti-GADPH (KM9002T) and anti-tubulin (KM9007T) antibodies were procured from Sanjian Biological.

### ShRNA and overexpression assay

The shRNA primers were obtained from NCBI, and the primer SN was TRCN0000278224. U2R and U2OS cells stably expressing shAHA1 were established by lentivirus infections with pLKO.1-shAHA1, and the control cells were infected with pLKO.1-Vector. U2R and U2OS cells stably expressing AHA1 and control cells were infected with pBabe-AHA1 and pBabe-Vector, respectively.

### Western blot (WB)

Cell lines or tumor tissues were harvested and lysed in RIPA buffer (1% NP-40, 0.5% sodium deoxycholate, 0.1% SDS, 10 ng/ml PMSF, 0.03% aprotinin, and 1 μM sodium orthovanadate) on ice for 30 min. After centrifugation for 10 min at 13,200 × rpm, the supernatants were collected, and the protein concentration was quantified by using Bradford assay. The proteins were separated on 10% SDS-PAGE gels and transferred to polyvinylidene difluoride membranes. The membranes were blocked with 5% BSA and incubated with the indicated primary antibodies, and corresponding horseradish peroxidase-conjugated secondary antibodies were used against each primary antibody. Proteins were detected using chemiluminescent detection reagents and films.

### MTT assay

Cell lines were cultured in a 96-well plate and treated with various concentrations of agents. After 3 days, 3-(4,5-dimethylthiazolyl-2)-2,5-diphenyltetrazolium bromide (MTT) was added to each well to a final concentration of 0.5 mg/ml. After incubation for 4 h, the resulting formazan crystals were dissolved in 100 μl of DMSO, and the absorbance was detected using a plate reader.

### Colony formation (soft agar) assay

Cell lines were harvested to obtain a density of 5 × 10^3^ cells/well. Then, 1% agar was melted in a microwave and cooled to 40 °C in a water bath, and 2X DMEM was warmed to 40 °C in a water bath and maintained for at least 30 min to allow the temperature to equilibrate. Equal volumes of the two solutions were mixed to obtain 0.5% agar + 2X DMEM, and 0.6 ml of this solution was then added to each well and allowed to set. Subsequently, 0.7% agar was melted in a microwave and cooled to 40 °C in a water bath, and 2X DMEM was also warmed to 40 °C in a water bath. For plating, 3 ml of 2X DMEM and 3 ml of 0.7% agar were added to a tube and mixed gently, and 0.6 ml of the resulting mixture was added to each replicate plate. The plate was then incubated at 37 °C in a humidified incubator for 10–14 days, and the resulting colonies were counted using a dissecting microscope.

### Transwell assay

U2R and U2OS cell lines were cultured at 1 × 10^4^ cells/200 μl in each Transwell® cell culture insert (8-μm pore size, 6.5-mm diameter; Costar, Cambridge, MA, USA) and allowed to migrate overnight. The Transwell® inserts were filled with 500 μl of Dulbecco’s modified Eagle’s medium supplemented with 10% fetal bovine serum. After incubation at 37 °C overnight, the filters were rinsed with PBS, fixed with methanol (10 min), and stained with crystal violet (0.1%) for 30 min. The cells on the upper surface of the filters were removed with a cotton swab, and the cells that migrated through the filters were counted under the microscope at a magnification of ×100. Each clone was tested in triplicate in at least two independent assays. The data are expressed as mean ± standard error of the number of cells obtained in each filter.

### Phalloidin staining assay

U2R and U2OS cell lines were seeded on glass coverslips for overnight and then fixed in 4% paraformaldehyde for 20 min and permeabilized with 0.1% Triton X-100 for 20 min at room temperature. The coverslips were incubated in the dark with 100 nM rhodamine-phalloidin at room temperature for 30 min. Nuclei were counterstained with 100 nM DAPI. The coverslips were rinsed in PBS and inverted on a drop of anti-fade mounting media on a glass slide. Then, these slides were sealed with neutral balsam and viewed under the confocal microscope.

### Nude mice tumor assay

BALB/c nude mice were obtained from the Guangdong Medical Laboratory Animal Center and fed amicrobic food and water. The care of mice was in accordance with institution guidelines. Five female nude mice aged 5 weeks and weighing 16–18 g were included in each group, and a subcutaneous osteosarcoma tumor model was established in these mice by subcutaneously injecting U2R-shControl and U2R-shAHA1 cells (2 × 10^6^ cells in 100 μl of DMEM) under the shoulder of the mice. Subsequently, an orthotopic model was then established in another set of mice. In this experiment, five female nude mice aged 6 weeks and weighing 16–18 g were included in each group, and U2R cells stably expressing shAHA1 or shControl were injected into the tibial medullary cavity of the right leg of the mice. The body weight of the animals and the two perpendicular diameters (*L* and *K*) of the tumor were recorded every 3 days.

The tumor volume (*V*) was calculated as

$$A = \frac{\pi }{6}\left( {\frac{{L + K}}{2}} \right)^3$$

### Immunohistochemistry (IHC) assay

IHC assay was performed based on a microwave-enhanced avidin-biotin staining method as previously described. Formalin-fixed, paraffin-embedded tumor tissue slides were deparaffinized using xylene and graded ethyl alcohol and then rinsed in water. Antigen retrieval was performed by boiling the slides in 0.01 M citrate buffer in a microwave oven for 5 min and cooling at room temperature. The slides were then incubated with 0.05% Triton X-100 in PBS for 5 min, and after the quenching of endogenous peroxides with 3% H_2_O_2_ in methanol, the slides were subjected to sequential treatments in a humidified chamber. The slides were blocked with 3% BSA for 30 min at room temperature, and then were incubated with anti-AHA1 polyclonal antibody A2617 and anti-Ki-67 rabbit monoclonal antibody overnight at 4 °C. The slides were subsequently incubated with the secondary rabbit-antibody for 30 min at room temperature. Next, the slides were stained successively with DAB dye for 5 min at room temperature, counterstained with hematoxylin, and coverslipped. The percentages of AHA1- and Ki-67-positive cells were quantified as the average from three fields in each slide. The expression of AHA1 and IDH1 was scored based on the intensity of staining and the percentage of positive cells. For the intensity of staining, 0 for negative, 1 for weak positive, 2 for moderately positive, and 3 for strong positive. For the percentage of positive cells, 0 for completely negative, 1 for 1–25% positive, 2 for 26–50% positive, 3 for 51–75% positive, and 4 for 76–100% positive. And the final score is the product of the score for the intensity of staining and the score for the percentage of positive cells. Patients with the final score 0–4 were classified as low expression, while patients with the final score higher than 4 were classified as high expression.

### Hematoxylin-eosin (HE) staining

After routine deparaffination, the sections were washed with double-distilled water and stained with hematoxylin for 7 min. The color was separated with 1% hydrochloric acid alcohol for 3–5 s. The sections were then dipped in distilled water for 30 s, dipped in 95% alcohol, and dyed with eosin for 1 min. Alcohol, dimethylbenzene, and neutral gum were separately and sequentially used for dehydration, hyalinization, and sealing. The sections were observed under a microscope at ×200 magnification.

### Analysis of metabolism

Cells were cultured in 35-mm culture dishes for 6 h or overnight, then the culture medium was replaced by fresh complete medium, and the cells were incubated for an additional 48 h. The media were collected for measurements of the glucose and lactate concentrations, and the cells were harvested to obtain protein lysates and measure GSH and ATP concentrations. The glucose levels were determined using a glucose assay kit (Nanjing Jiancheng), and glucose consumption was calculated by deducting the measured glucose concentration in the media from the original glucose concentration. The lactate levels were determined using a lactate assay kit (Nanjing Jiancheng). The intracellular GSH and ATP levels were measured using a glutathione assay kit (Nanjing Jiancheng) and an ATP concentration assay kit (Nanjing Jiancheng), respectively. All the values were normalized based on the total protein concentration obtained using the Bradford protein assay.

The levels of ECAR and OCR were assessed using an XFe96 Analyzer (Seahorse Bioscience). Cells were cultured in a Seahorse XF cell culture microplate at a density of 2 × 10^4^ cells/well (U2R) or 1 × 10^4^ cells/well (U2OS).

### Co-immunoprecipitation (Co-IP)

Cells were lysed in lysis buffer (1% Nonidet P-40, 150 mM NaCl, 100 mM HEPES, 5 mM Na_4_P_2_O_7_, 5 mM NaF, 2 mM Na_3_VO_4_, 1 mM phenylmethylsulfonyl fluoride, 10 mg/l aprotinin, and 10 mg/l leupeptin), and cleared cell lysates were incubated with Protein G-conjugated Sepharose or Protein A-conjugated Sepharose (GE Healthcare, Piscataway, NJ, USA) and the appropriate antibody overnight at 4 °C. Following incubation, the resin was washed three times with co-immunoprecipitation lysis buffer, and protein samples were eluted by boiling in 5 X SDS sample buffer for western blotting analysis.

### Gateway assay

Gateway upstream and downstream primers of IDH1 were designed for Polymerase Chain Reaction (PCR). The IDH1 PCR products were then connected to pDONR223 by BP Clonase^TM^ II for 6 h at 25 °C. pDONR223-IDH1 was identified by BsrG1-enzyme digestion. pDONR223-IDH1 was associated with the pCDH-Neo-Flag-Venus/Vector by incubation with LR Clonase^TM^ II for 6 h at 25 °C, and pCDH-Neo-Flag-Venus/IDH1 was also identified by BsrG1-enzyme digestion.

### Statistical analyses

Student’s *t*-test was used to compare individual data points between two groups. Comparisons among three or more groups were performed using one-way ANOVA test. Pearson correlation coefficient was calculated to show the relationship between the expression levels of AHA1 and IDH1 protein. The Kaplan–Meier method and the log-rank test were used to compare patient survival. Data are presented as mean ± SD or median with interquartile range. In all analyses, *p* < 0.05 was considered to indicate statistical significance.

## Supplementary information

SIGTRANS-00923R1_Supplementary_Materials

## Data Availability

The datasets used and/or analyzed during the current study are available from the corresponding author on reasonable request.
