# Supplementary material for: AHA1 upregulates IDH1 and metabolic activity to promote growth and metastasis and predicts prognosis in osteosarcoma
Source: Signal Transduct Target Ther. 2021 Jan 20;6:25. doi: 10.1038/s41392-020-00387-1 (PMC7815748; doi:10.1038/s41392-020-00387-1)
Supplement: Supplementary file 1 — SIGTRANS-00923R1_Supplementary_Materials [file 41392_2020_387_MOESM1_ESM.docx]

Supplementary Materials for

AHA1 upregulates IDH1 and metabolic activity to promote growth and metastasis and predicts prognosis in osteosarcoma

Diwei Zheng, Weihai Liu, Wenlin Xie, Guanyu Huang, Qiwei Jiang, Yang Yang, Jiarong Huang, Zihao Xing, Mengling Yuan, Mengning Wei, Yao Li, Junqiang Yin, Jingnan Shen, Zhi Shi

Correspondence to: yinjunq@mail.sysu.edu.cn, shenjn@mail.sysu.edu.cn, tshizhi@jnu.edu.cn

**This PDF file includes:**

Supplementary Materials and Methods

Supplementary Figures. S1 to S7

Supplementary Tables S1 to S3

Supplementary Materials and Methods

Wound healing assay

All cell lines were cultured in a 24-well plate at a density of 6 × 104 cells/well. An injury was created using a 2-mm-wide plastic pipette tip. After incubation, the excess liquid in the wells was removed, and the wells were rinsed with PBS and covered with serum-free medium. The cells were then incubated for 48 h. The cell migration area was quantified by analyzing photographic images acquired at different time points (0 h, 24 h, and 48 h).

Overexpression assay of synonymous AHA1

To generate synonymous AHA1 (WT-AHA1 of ShRNA targeting sequences: gtccctgagaaacatattgtg; synonymous AHA1: …gtAccAgaAaaGcaCatCgtT…) were synthesized and subcloned in frame into lentiviral vetors containing expression F2A-GFP. U2R-shAHA1 and UO2S-shAHA1 cells were overexpressed the synonymous AHA1-GFP or GFP as the negative control through lentivirus infection.

In Vitro Gene Silencing

IDH1 siRNA and the negative control were purchased from RiboBio (Guangzhou, China) and transfected into osteosarcoma cells using Lipofectamine 2000 (Life Technologies, Carlsbad, CA, USA) according to the manufacturer’s instruction.

Figure. S1. Upregulation of AHA1 in osteosarcoma is not correlated with age, sex, primary location or tumor size


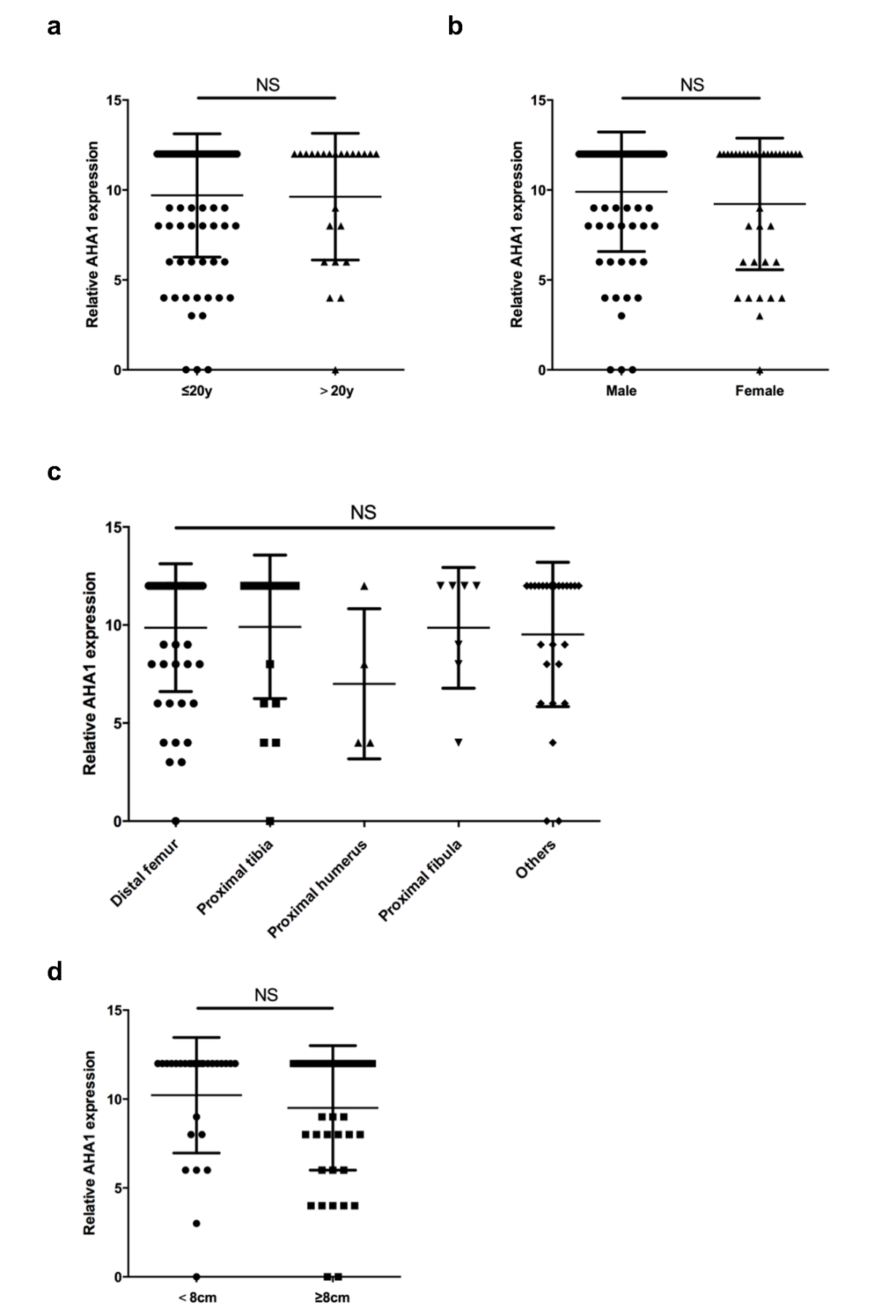


**Supplementary Fig. S1** Upregulation of AHA1 in osteosarcoma is not correlated with age, sex, primary location or tumor size. The relative expression levels of AHA1 in osteosarcoma stratified by age (**a**), sex (**b**) and tumor size (**d**) were analyzed with unpaired t-test. The relative AHA1 expression in osteosarcoma stratified by primary location (**c**) was analyzed with one-way ANOVA test. Data are presented as mean ± SD. NS: no statistical significance.

Figure. S2. AHA1 is important for migration of osteosarcoma cells *in vitro*


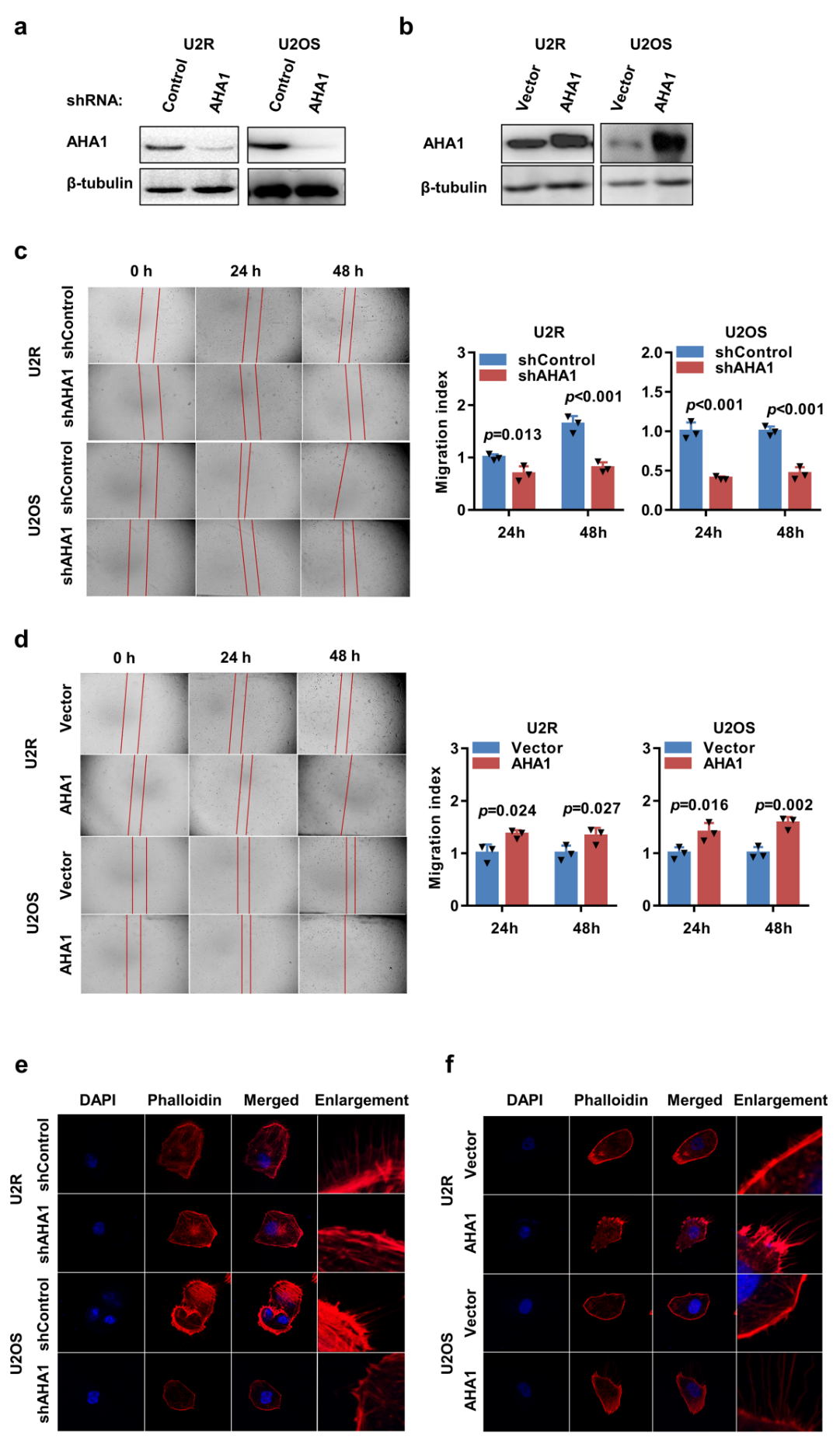


**Supplementary Fig. S2** AHA1 is important for migration of osteosarcoma cells *in vitro*. **a** WB showing the AHA1 knockdown efficiency in U2R and U2OS cells compared with that in cells stably expressing nonspecific shRNA (shControl). **b** WB showing the overexpression of AHA1 in U2R and U2OS cells compared with cells expressing FLAG-Vector. **c, d** Wound healing assay of U2R and U2OS cells. Representative images obtained at 0 h, 24 h and 48 h are shown. Data are shown as mean ± SD of three independent cultures. **e, f** Cells were subjected to F-actin (red) and DAPI (blue) staining and analyzed under an immunofluorescence microscope.

Figure. S3. AHA1 is important for drug resistance of osteosarcoma cells *in vitro*


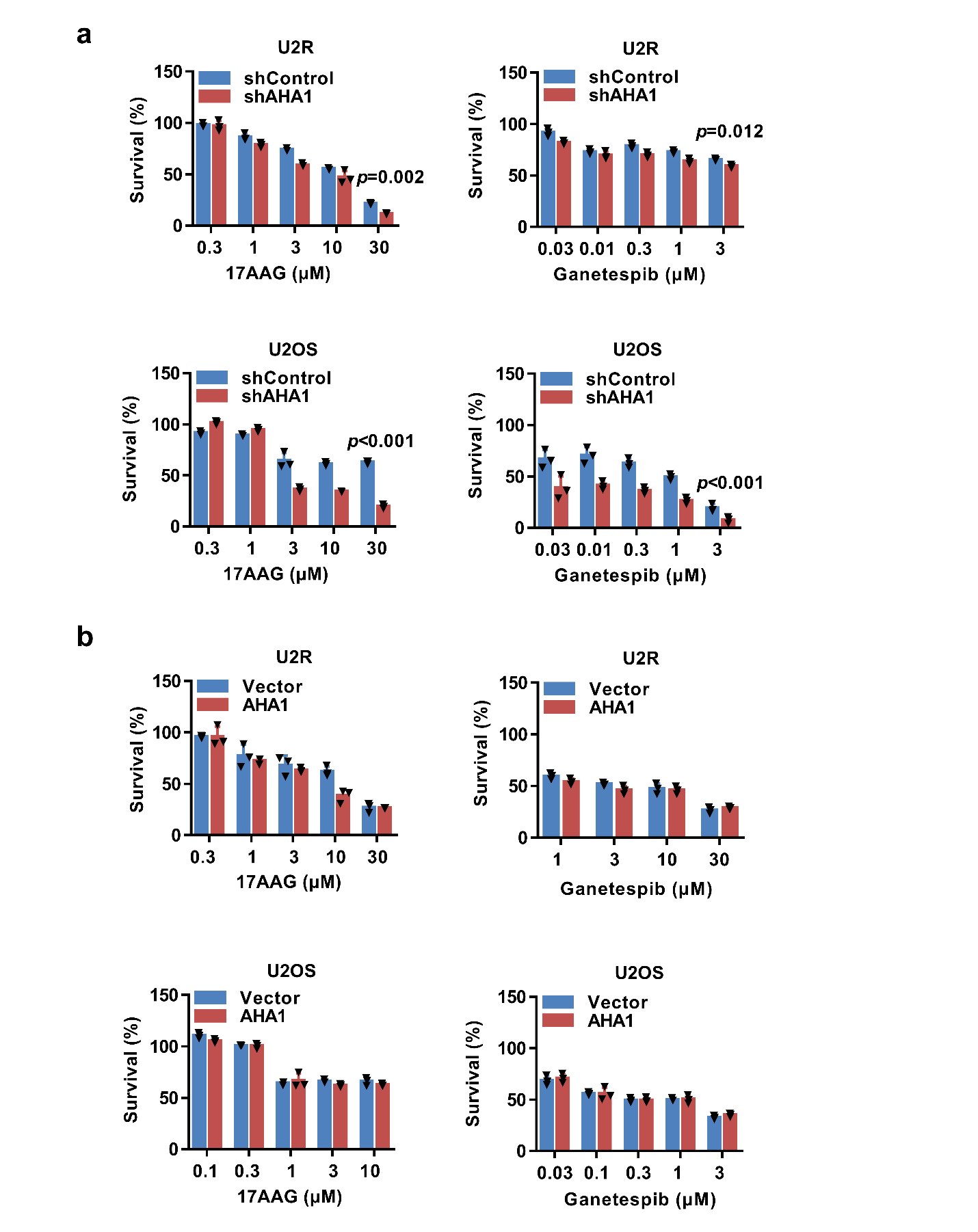


**Supplementary Fig. S3** AHA1 is important for drug resistance of osteosarcoma cells *in vitro*. **a** The sensitivities of U2R and U2OS cells stably expressing shControl or shAHA1 to the Hsp90 inhibitors 17AAG and ganetespib are shown. **b** The sensitivities of U2R and U2OS cells stably expressing the control vector or AHA1 to the Hsp90 inhibitors 17AAG and ganetespib are shown. **a, b** Data are shown as mean ± SD of three independent cultures.

Figure. S4. Overexpression of AHA1 rescues the effect of AHA1 knockdown on the growth and migration of osteosarcoma cells *in vitro*


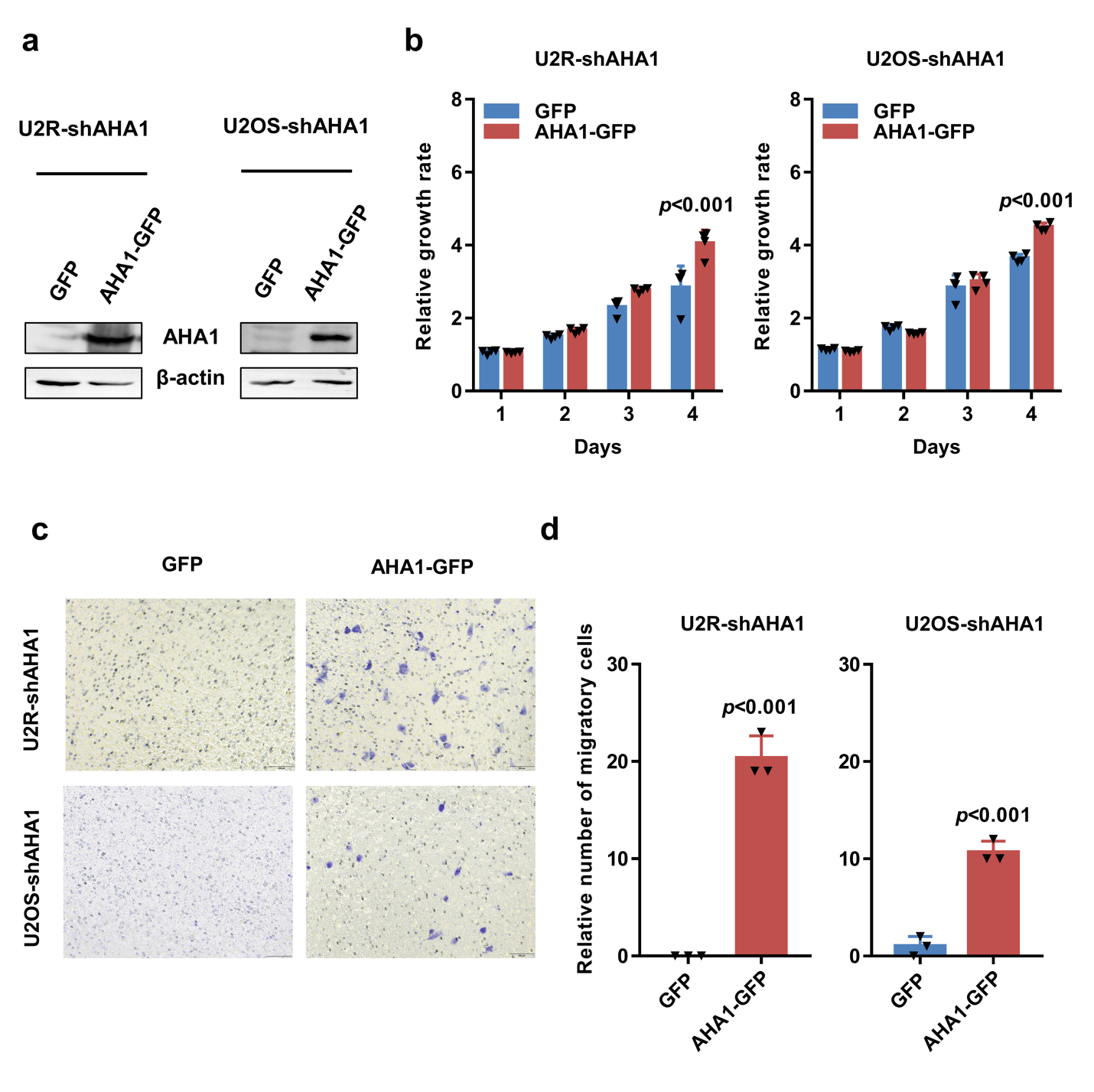


**Supplementary Fig. S4** Overexpression of AHA1 rescues the effect of AHA1 knockdown on the growth and migration of osteosarcoma cells *in vitro*. **a** WB showing the overexpression of AHA1 in U2R-shAHA1 and U2OS-shAHA1 cells. **b** Proliferation was examined by MTT assay. **c, d** Cells were cultured in the Transwell® cell culture insert, stained with 1% crystal violet, and observed under a fluorescence microscope. The number of cells that traversed the filters was counted, and triplicate samples were analyzed. **b** Data are shown as mean ± SD of four independent cultures. **d** Data are shown as mean ± SD of three independent cultures.

Figure. S5. AHA1 is critical for metabolic activity


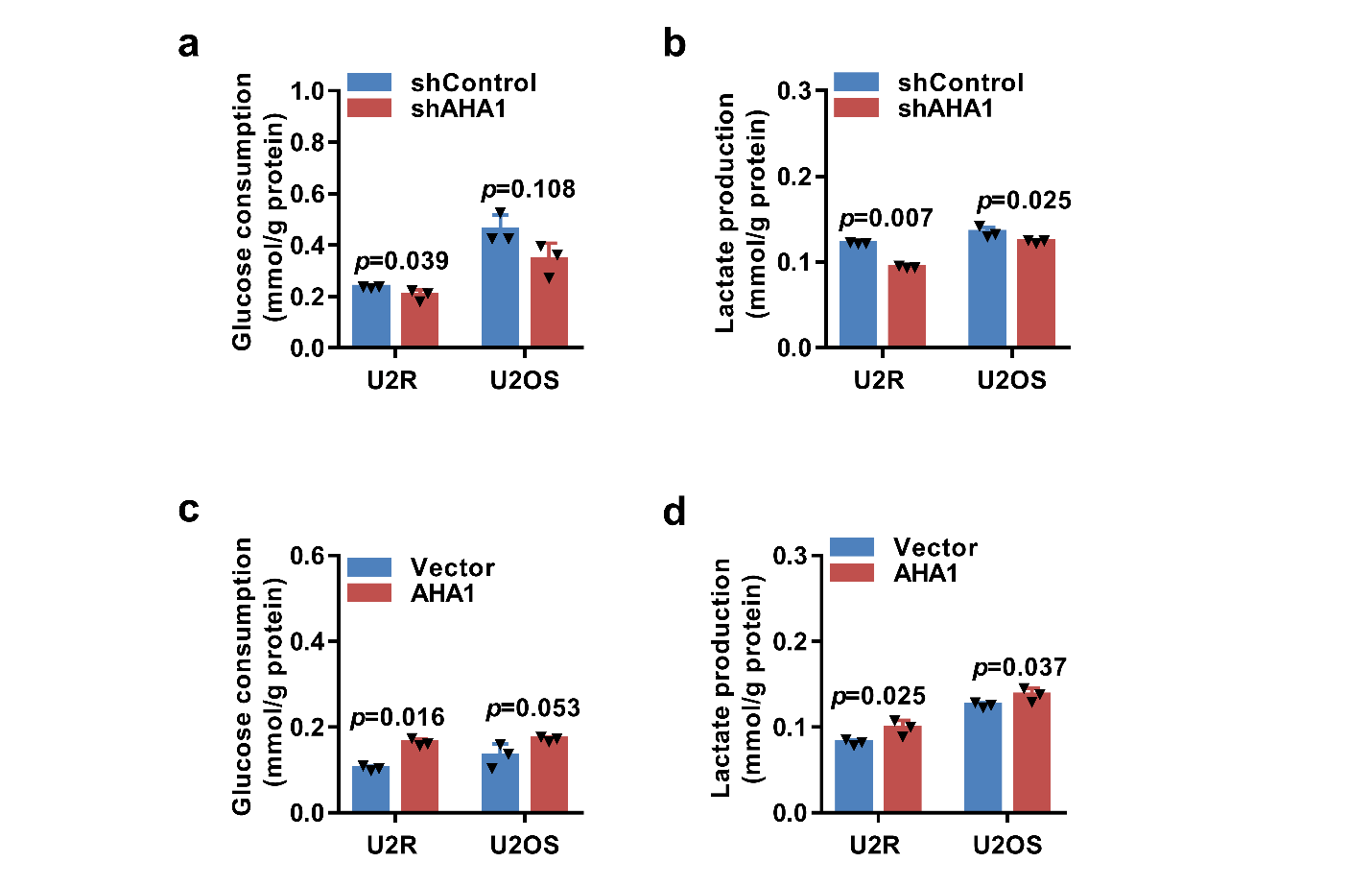


**Supplementary Fig. S5** AHA1 is critical for metabolic activity. **a-d** Glucose consumption and lactate production of U2R and U2OS cells with AHA1 knockdown or overexpression are shown. **a-d** Data are shown as mean ± SD of three independent cultures.

Figure. S6. IDH1 knockdown enhances the effect of AHA1 knockdown on the growth, migration and drug sensitivity of osteosarcoma cells *in vitro*


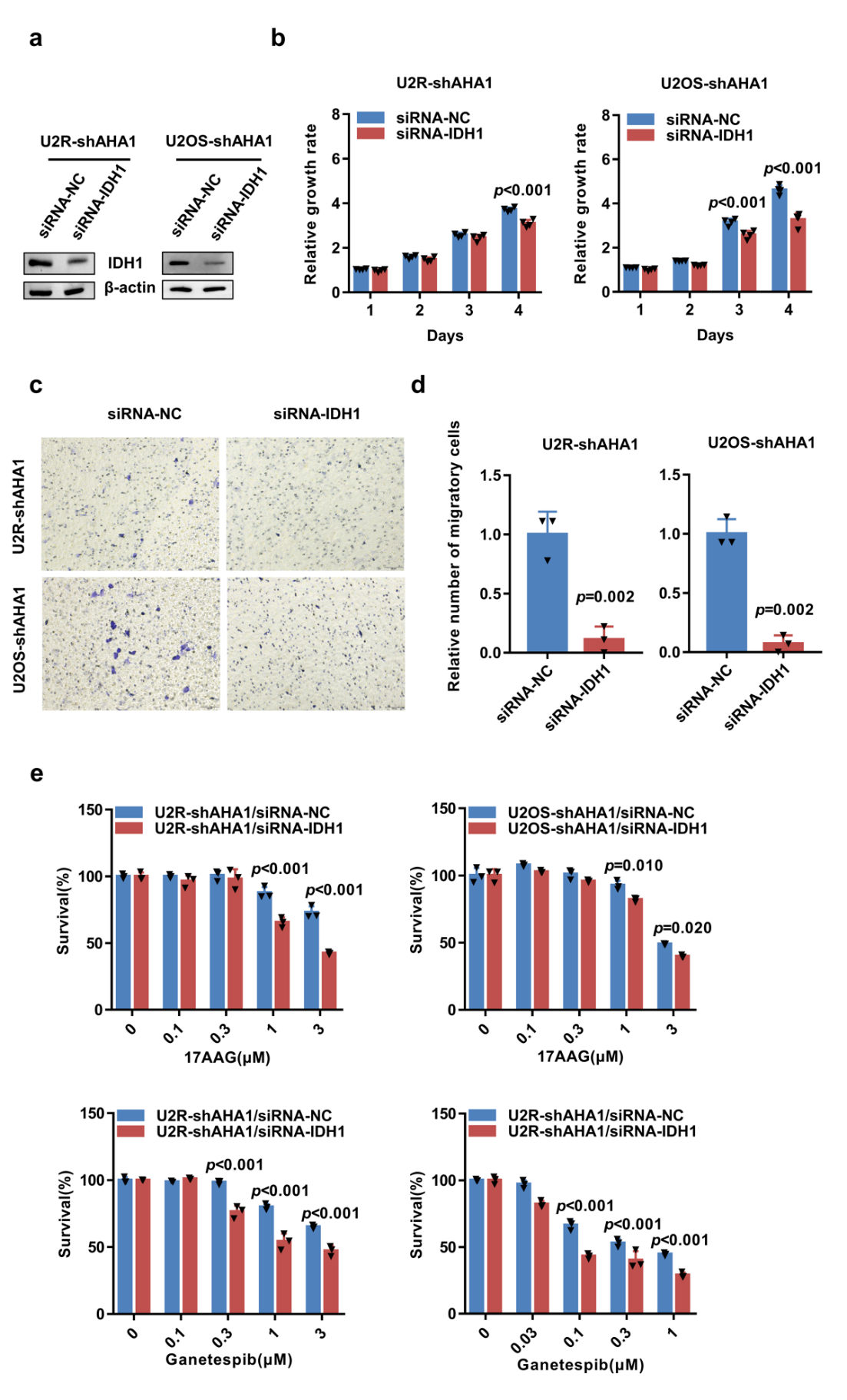


**Supplementary Fig. S6** IDH1 knockdown enhances the effect of AHA1 knockdown on the growth, migration and drug sensitivity of osteosarcoma cells *in vitro*. **a** WB showing the IDH1 knockdown efficiency in U2R-shAHA1 and U2OS-shAHA1 cells compared with that in cells transfected with nonspecific siRNA (siRNA-NC). **b** Proliferation was examined by MTT assay. **c, d** Cells were cultured in the Transwell® cell culture insert, stained with 1% crystal violet and observed under a fluorescence microscope. The number of cells that traversed the filters was counted, and triplicate samples were analyzed. **e** The sensitivities of U2R-shAHA1 and U2OS-shAHA1 cells with siNC or siIDH1 to the Hsp90 inhibitors 17AAG and ganetespib are shown. **b** Data are shown as mean ± SD of four independent cultures. **d, e** Data are shown as mean ± SD of three independent cultures.

Figure. S7. Upregulation of IDH1 in osteosarcoma is not correlated with age, sex, primary location or tumor size


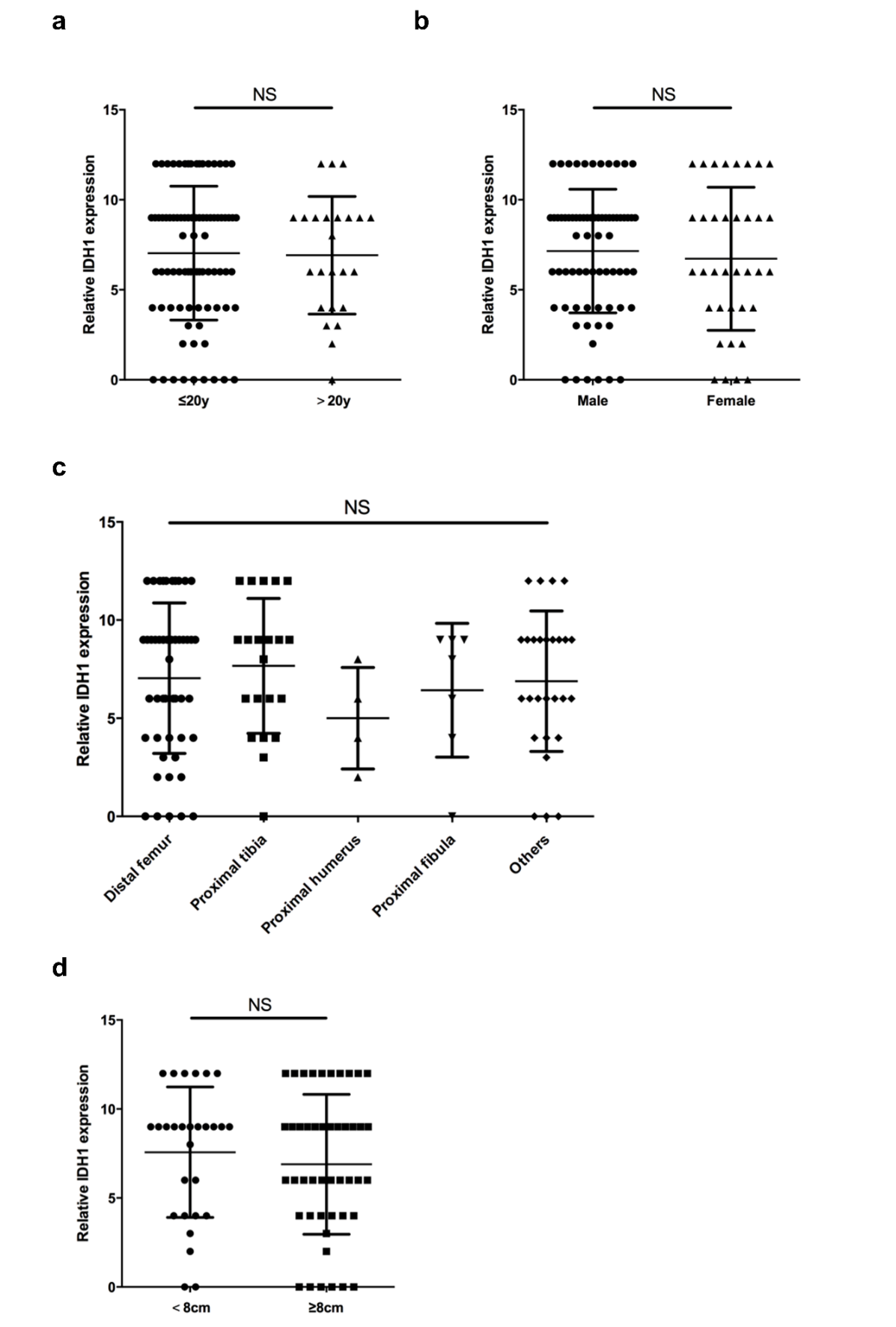


**Supplementary Fig. S7** Upregulation of IDH1 in osteosarcoma is not correlated with age, sex, primary location or tumor size. The relative expression levels of IDH1 in osteosarcoma stratified by age (**a**), sex (**b**), and tumor size (**d**) were analyzed with unpaired t-test. The relative IDH1 expression in osteosarcoma stratified by primary location (**c**) was analyzed with one-way ANOVA test. Data are presented as mean ± SD. NS: no statistical significance.

Table S1. The association of AHA1 and IDH1 expression with the prognosis of osteosarcoma patients

|  | AHA1 level | | *P* value* | IDH1 level | | *P* value* |
| --- | --- | --- | --- | --- | --- | --- |
|  | High | Low |  | High | Low |  |
| Lung metastasis |  |  | 0.032 |  |  | 0.073 |
| Yes (57) | 53 | 4 |  | 45 | 12 |  |
| No (52) | 41 | 11 |  | 33 | 19 |  |
| Death |  |  | 0.021 |  |  | 0.014 |
| Yes (52) | 49 | 3 |  | 43 | 9 |  |
| No (57) | 45 | 12 |  | 35 | 22 |  |

*Chi-square test

Table S2. The association of AHA1 expression with IDH1 expression

|  | AHA1 | | *P* value* |
| --- | --- | --- | --- |
|  | High | Low |  |
| IDH1 |  |  | 0.009 |
| High | 72 | 6 |  |
| Low | 22 | 9 |  |

*Chi-square test

Table S3. Multivariate analysis of overall survival in 109 osteosarcoma patients

|  | Variable | *RR* | 95% *CI* | *P* value* |
| --- | --- | --- | --- | --- |
| Overall survival |  |  |  |  |
|  | Enneking stage | 22.682 | 19.699-26.117 | 0.000 |
|  | AHA1 | 1.533 | 1.303-1.805 | 0.000 |
|  | IDH1 | 1.853 | 1.561-2.199 | 0.000 |

*Cox proportional hazards regression
